# Implementation of a problem-solving training initiative to reduce self-harm in prisons: a qualitative perspective of prison staff, field researchers and prisoners at risk of self-harm

**DOI:** 10.1186/s40352-019-0094-9

**Published:** 2019-07-31

**Authors:** Amanda E. Perry, Mitch G. Waterman, Allan O. House, Joanne Greenhalgh

**Affiliations:** 1 Department of Health Sciences, University of York, Fulford, York, YO10 5DD UK; 2 Department of Psychology, University of Leeds, Leeds, LS2 9JT UK; 3 Leeds Institute of Health Sciences, University of Leeds, Leeds, LS2 9JT UK; 4 Sociology and Social Policy, Faculty of Education and Social Science and Law, University of Leeds, Leeds, LS2 9JT UK

**Keywords:** Prison, Qualitative, Problem-solving, Self-harm, Training

## Abstract

**Background:**

Social problem-solving is one technique used to help reduce incidence of self-harm. Our study evaluated the feasibility and acceptability of the adaptation and implementation of a brief Problem-Solving Training (PST) intervention to reduce self-harm in prisons.

**Methods:**

The process involved i) adaptation of the training materials using focus groups with prison staff and prisoners, ii) training frontline prison staff to use the skills, and iii) implementation of the skills with prisoners at risk of self-harm. Qualitative interviews were conducted with prison staff, prisoners and field researchers and were analysed using a thematic framework to produce a model of the barriers and facilitators to the process.

**Results:**

We conducted 43 interviews across three prison sites. The interviews included 19 prison staff, 18 prisoners and six field researcher meetings. The adaptation to the training and intervention materials were well received. The findings identified the need to support training using a collaborative and flexible approach. Prisoner engagement was affected by their own personal circumstances and by a range of contextual issues relating to the prison environment. Implementation of the skills by prison staff were hindered by resource constraints, the prison environment and staff attitudes.

**Conclusions:**

We found that it was feasible to adapt an existing intervention and contextualise it within the prison environment. Although we could train large numbers of staff it was deemed unfeasible for staff to implement the problem-solving skills to prisoners at risk of self-harm. Prisoners who engaged with the intervention reported a range of benefits. Alternative implementation mechanisms to tackle the contextual barriers proposed by staff and prisoners included delivery of the intervention using an educational setting and/or use of a prisoner peer-led scheme.

**Electronic supplementary material:**

The online version of this article (10.1186/s40352-019-0094-9) contains supplementary material, which is available to authorized users.

## Background

Self-harm in UK prisons has risen over the last 5 years (Ministry of Justice, 2016) and represents a worldwide public health problem (WHO, 2014). Terms relating to self-harm (e.g., self-injury, self-injurious behaviour, self-mutilation, deliberate self-harm, deliberate self-injury, non-suicidal self-injury, self-cutting, self-mutilation behaviour and para-suicide) refer to the notion of a self-harm event, regardless of the individual’s intent and motivation. Self-harm is also often associated with suicide, and persons including suicide attempt, suicidal behaviour, suicidal gesture and suicide ideation and/or self-inflicted death were included in the study.

In the UK, prison staff use a safeguarding process referred to as ACCT (Assessment, Care in Custody and Teamwork) to monitor prisoners who self-harm or attempt suicide. This process involves a series of assessments followed by the development of a care map plan, providing the prisoner with additional support (see http://www.ppo.gov.uk/app/uploads/2014/07/ACCT_thematic_final_web.pdf). Whilst improvements in practice continue to develop, access to psychological therapies and additional ways of helping individuals at risk of self-harm are required to support the ACCT procedure (Forrester & Slade, 2014). Identifying ways to reduce self-harm is particularly important given the increased likelihood of suicide (Hawton, Linsell, Adeniji, Sariaslan, & Fazel, 2014). However, supporting prisoners at risk of self-harm is complex and challenging in an environment which has the simultaneous responsibility for the punishment, rehabilitation and health of people under its care.

Previous randomised controlled trials aimed at reducing self-harm in prisons have included the use of Cognitive Behavioural Therapy (CBT) and individual psychotherapy sessions (Pratt et al., 2015; Walker, Shaw, Turpin, Reid, & Abel, 2017). Despite encouraging findings, these interventions require trained clinical staff to employ up to 20 therapy session (sometimes twice weekly, lasting an hour in length to prisoners). Any such approach may therefore exclude prisoners from accessing treatment if they are on short-term sentences or subject to transfer to another prison.

Furthermore, in the current context, UK prisons have experienced reductions in budgets and staff redundancies, leaving them to manage the running of the prison with limited resources and staff shortages. It is therefore necessary to explore how prison staff can enable the reduction of self-harm using a briefer evidence-based intervention. This principle supports previous UK policy initiatives which over time have shifted the medicalisation of self-harm to a position where *‘Suicide is Everyone’s Concern*’ (HMIP, 1999). It also recognises a series of research recommendations that calls for staff to be adequately trained to deal with the management and prevention of self-harm, ((Walker et al., 2017) see National Institute of Clinical Excellence (NICE) Guidance research recommendations for the long-term management of self-harm: https://www.nice.org.uk/guidance/cg133/chapter/2-Research-recommendations).

The theoretical underpinning for social problem-solving originally stems from a concept outlined by D’Zurilla in 1971 who defined the problem-solving process as a self-directed cognitive behavioural approach in which a person attempts to identify or discover effective or adaptive ways of coping with problematic situations (D’Zurilla & Goldfried, 1971; Evans et al., 1999). Since then, other researchers have added to this pivotal work both theoretically and empirically (e.g., Daunic, Smith, Garvan, Barber, Becker, Peters & Naranjo, 2012). The process of problem-solving typically involves between 5 and 7 recognised steps including (i) identifying that a problem exists, (ii) defining the problem, (iii) generating solutions, (iv) evaluating the solution using pros and cons, (v) creating an action plan and (vi) reviewing the outcome. Individuals who self-harm can often struggle to use social problem-solving skills (D’Zurillia 1998), resulting in reliance on others and use of passive (as opposed to a proactive) approaches to problem-solving (Linehan et al., 1987; McLeavey et al., 1994; Pollock & Williams, 2011).

Problem solving skills have been used in a variety of different contexts and are promoted by The World Health Organisation as ‘Problem Management Plus’ (PM+) (WHO, 2016). The initiative was devised as a psychological intervention that could be quickly learned not only by professionals but also by people who are not mental health trained. They refer to their scheme as a simplified, scalable intervention, in that their delivery requires a less intensive level of specialist human resource (Sijbrandij, Farooq, Bryant, Dawson et al., 2015). They use the term “problem management” rather than “problem-solving” because they argue that some people are likely to face many problems that may be difficult to solve. For example, individuals experiencing war, communal violence or chronic poverty may have little or no control over such problems (WHO, 2016). Similarities may also be displayed by people experiencing imprisonment.

Evaluations of problem-solving skills using randomised controlled trials in the *community* show promising results, but are yet to be tested in the prison environment (Hawton et al., 2016; Perry, Waterman, & House, 2015). For this reason, the feasibility of these techniques within the prison environment need to be explored before enabling an evaluation of effectiveness (see: https://mrc.ukri.org/complexinterventions-guidance/). Our study therefore sought to: 1) adapt an existing community-based problem-solving skills intervention for use within the prison, 2) deliver training to prison staff, and 3) for staff to implement the skills with prisoners at risk of self-harm. The process involved co-producing the materials with Her Majesty’s Prison and Probation Service (HMPPS) staff, prisoners and the research team to devise an approach that was context specific and relevant to those that were using it. This approach is supported by those who have increasingly called for more explicit attention to facilitate partnerships between professionals and the beneficiaries of public health services (Alford & Yates, 2015; Pestoff, 2009; Radnor, Osbourne & Kinder, 2014). Here we report on the qualitative research findings from the study, which assess the adaptation and delivery of the staff training package, and the implementation of the intervention to prisoners at risk of self-harm. The quantitative findings from the wider study are reported elsewhere (see Perry et al. in press 2019).

## Methods

### Study design and setting

The study used a mixed methods design to assess the feasibility and acceptability of the Problem-Solving Training (PST) intervention in four UK prisons in the Yorkshire and Humber region between September 2014 and May 2017. The study sites included two male adult local prisons where most prisoners were awaiting sentence (housing up to 1212 and 1052 prisoners, prisons A and B), one female prison (housing up to 416: prison C) and one male resettlement prison where sentenced prisoners are housed prior to transfer or release into the community (housing up to 825: prison D). Ethical approval for the study was obtained for each phase of the study.

### The original intervention

The problem-solving intervention was originally devised in New Zealand for people who self-harm in the community and was chosen because of its subsequent evaluations using evidence from randomised controlled trials in New Zealand and also in UK hospital emergency departments (Collinson et al., 2014). The seven-step model includes getting the right attitude (step one), reflection and recognising triggers (step two), defining a clear problem (step three), brain storming solutions (step four), decision making (step five), making a plan (step six) and reviewing progress (step seven).

#### The adaptation of the training and intervention materials

During 2015, the adaptation from the original intervention was completed using a sample of nominated prison staff and prisoners who took part in a series of focus groups. The focus groups were used to: (i) ensure the appropriateness and context of the case materials and (ii) to promote discussion with staff and prisoners about how they thought the training might be implemented. Thirty-one prison staff attended the focus groups. The groups comprised of operational 17/31(34%), managerial 6/31 (12%), healthcare 3/31 (6%), external agency staff 2/31 (4%), probation and administration 3/31 (5%) staff with a mean age of 37 years (SD 13.16). The majority were female 20/31 (66%), spoke English as their first language 27/31 (88%) and were British 27/31 (90%). Six focus groups involving 67 (mainly male) prisoners, 56/67 (83.6%) with a mean age of 39.8 years (SD 9.63) engaged with the process that resulted in two gender-specific picture booklets that were used in the training and delivery of the intervention and a series of exercises with associated case study scenarios see example in Additional file 1 (Perry et al., 2015). It was intended that the entirety of the intervention would be delivered using a single 30 min session to reduce attrition but also to support the use of a brief intervention that could be implemented by any member of staff within the constraints of the organisation.

#### Recruitment and training of frontline prison staff

Frontline staff were recruited with the help of prison representatives who assisted with room bookings and detailing individuals according to shift patterns to attend the training course. We wanted to take a holistic approach to providing training for staff and eligible staff included anyone with responsibility for prisoners at risk of self-harm. Invited staff groups included management, probation, teaching, prison officers, chaplaincy, psychologists, specialist suicide prevention assessors and nursing staff. The training consisted of a one-hour session, which took place between March 2015 and August 2016. Training was delivered by the research team in a flexible manner (e.g., during induction or on a lunchtime). All staff receiving the training gave full informed consent.

Two hundred eighty frontline prison staff across 4 prisons were trained by the research team with a mean of 8 staff per training group (range 2–19). Recruitment of staff to training sessions appeared to be acceptable and feasible and we exceeded our anticipated training goal (*n* = 125). Staff trained were mainly operational prison officers (120/280 43%) but the training was also attended by, healthcare staff (78/280 28%), voluntary, managerial, administrative, educational, and offender manager probation staff (82/280 29%). The mean age of staff trained was 42 years, 59% were male, and almost all spoke English as their first language and were British. Trained staff had spent a median of 8 years (range < 1 month – 36 years) working in the prison service.

#### Recruitment and delivery of problem-solving skills to prisoners at risk of self-harm

Recruitment of prisoners occurred at prison sites A, B and D. In site C access to the prison was limited and delivery of the intervention did not occur as intended. Prisoners at all other sites were identified using an ‘at risk’ register and approached by a member of the research team or prison staff.

Eligible prisoners were: 1) > 16 years and (2) had an episode of self-harm or attempted suicide in the previous 2 weeks. Prisoners were excluded if: an ACCT was opened for reasons other than actual self-harm, they were deemed too unwell by prison staff, or if they posed a risk to the researchers. The original study design conceived that staff would cascade the problem-solving skills to prisoners ‘at risk’. Through talking to staff about their experiences of trying to implement the intervention it became apparent that this was not feasible for a number of different reasons. Staff were found to implement the training for two of the 48 prisoners who were recruited to the study. As part of the study risk analysis plan it was subsequently decided that this task would be taken over by the research team who delivered the intervention with the remaining 46 prisoners.

The median length of time spent on intervention delivery across one session was 40 min per prisoner, (range 30–90 min). The overall time spent with the researcher, including providing information about the study and gaining informed consent, conducting the baseline assessment, intervention delivery; administering the follow up questionnaires and conducting the qualitative interviews averaged a median of 80 min, (range 30 min up to 2 h 30 min). The total process included up to seven appointments with all prisoners receiving the initial intervention delivery session. Some prisoners requested follow-up appointments to support their use of the intervention booklets and materials (30/48 62%).

### The evaluation

#### Qualitative interviews

We intended to sample 30 staff and 10 prisoners (across the three sites) and capture the experiences of the field researchers during a series of planned team meetings. The interviews were used to identify the perspectives of staff and prisoners on the feasibility and acceptability of the adapted materials, the training sessions and the implementation of the intervention. The semi-structured interview schedule for staff included a range of different topics to understand more about the feasibility of conducting training sessions in a prison environment and the implementation of the problem-solving skills with prisoners at risk of self-harm. The interviews were broadly structured into the following topic areas: (i) an examination of the organisation requirements to train, (ii) format of the training sessions and the materials to be used to support the training, (iii) the training methodology used across the prison sites and (iv) staff delivery of the intervention to those at risk of self-harm.

The semi-structured interview schedule for prisoners was like that used by staff but also included a fuller exploration of using the problem-solving skills in the prison environment. The schedule included the following topics: (i) delivery of the intervention by the research team, (ii) the interplay of the prison environment and the intervention, (iii) barriers to engagement with the intervention, (iv) factors that improved engagement with the intervention, (iv) mechanisms for how the intervention worked and (v) the impact of the intervention on self-harm.

The research team approached staff and prisoners consecutively to see if they were willing to take part in an interview. We intended to collect data from staff and prisoners who did not attend the training, but this proved not feasible. We were granted permission to use a tape recorder in two of our three sites. We recorded (where possible) anonymous interviews using participant identification numbers. Where recording was not permitted, we took verbatim notes and verified these with the participant at the end of the interview. Interviews lasted up to an hour and on one occasion a group of prisoners and staff were interviewed together. Prisoner interviews were mainly conducted in prison healthcare department and staff interviews were conducted either at the person’s place of work or over the telephone at a pre-designated time.

The field researchers met periodically throughout the project to reflect on how the training and intervention were perceived to be working in each site. These sessions were recorded and transcribed to provide an additional perspective on the mechanisms underlying the implementation of the training, intervention delivery and acceptability.

### Data analysis plan

The transcripts were analysed by an independent researcher who had not been involved in the delivery of the intervention. Drawing on a realist philosophy of science, it was assumed that interventions are never universally successful, and the mechanisms through which they work are heavily shaped by the design of the intervention itself and the context into which the intervention is implemented (Pawson & Tilley, 1997). The analysis therefore sought to understand how the prison context, the social circumstances of the prisoners and the design of the intervention shaped the ways in which prisoners responded to the resources offered by the intervention. The interview transcripts were imported into Nvivo version 10 (see: https://www.qsrinternational.com/nvivo/nvivo-products) and were coded line by line, the development of descriptive themes were grouped by codes based on their similarities and generation of higher level themes based on our research aims: (i) adaptation of the training materials, (ii) training staff to deliver the problem-solving skills and, (iii) implementation of the problem-solving skills with prisoners at risk of self-harm. Within each aim we identified ‘lower level’ themes that emerged from the data that were collated into a model of how the intervention was adapted, how staff were trained and how the intervention was implemented which summarised the facilitators and barriers to each section of the study process.

## Results

### Interview sample

**Fig. 1:**
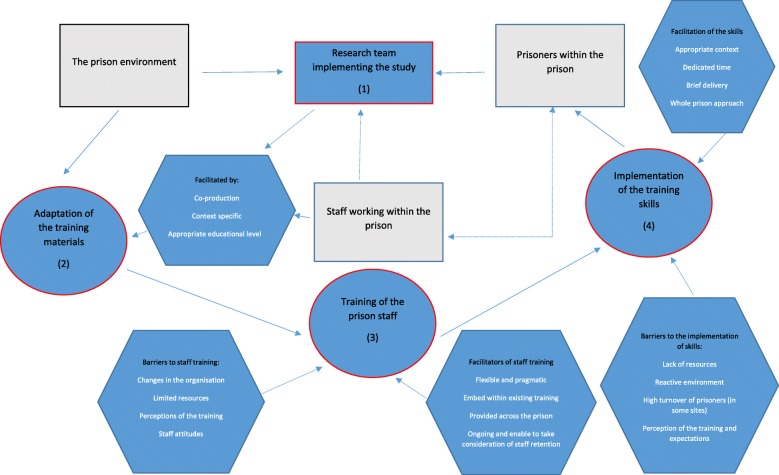
Framework model summarising the facilitators and barriers linked to the adaptation, training and implementation of a problem-solving training skills package for prison staff and prisoners at risk of self-harm behaviour

We conducted 43 interviews across our three prison sites. The interviews included 15 frontline staff representing healthcare staff (*n* = 5), staff involved with the ACCT process (*n* = 6), senior operational staff on the prison wings (*n* = 4) and chaplaincy staff (*n* = 4). Eighteen prisoners were interviewed, and six meetings were recorded with the field researchers. Figure 1 summaries the model produced from the evidence to show the key barriers and facilitators to each element of the training implementation and intervention delivery. The shapes outlined by the red boundary represent the three main study aims.  These were informed by three elements; the prison environment, prisoners within the prison and the staff working within each prison site.  At each stage within the project barriers and facilitators were grouped together in this diagram to help summarise the findings across the qualitative interviews described below.

#### Stage 2: Adaptation of materials and training package

Emerging lower level themes around the adaptation of the materials and training package included the importance of adaptation through co-production, the necessary requirement to generate materials that are perceived as being relevant to the context and environment in which they were used:

*‘Well, when I first got the booklet I thought, oh no, here we go again, it was another self-help style booklet. But when I’ve read it, the fact that it relates to somebody who I could associate with because they’re in a similar environment’ PRISONER*

Other research has shown that this process determines its worth in whether individuals use the intervention within the system and can help to support the suggestion that failure to recognise the unique character of an organisation and its implications might limit the success in collaborating with frontline prison staff and prisoners to improve healthcare (Batalden et al., 2017).

Literacy levels within the prisoner population are poor in comparison to the general population and the booklets we produced contained a number of pictures and stories that helped to facilitate the skills we were trying to teach. One prisoner commented that the pictures were a helpful element of the booklets and facilitated them to understand the skills that were being presented: “*They are good. For someone who couldn’t read and write or showing they couldn’t understand, positive, negative, just from a picture which is just simple. It was good, yes*”. As such, participants felt the booklet would be suitable for ‘*all sorts’* of people.

The co-production of the adaptation process also identified potential barriers, which might prevent engagement (see Fig. 1). For example, we found participants readily able to relate, define and identify problems but struggle to find solutions to their problems. One of the challenges of problem-solving in an environment where resources are necessarily constrained is that problem solving becomes necessarily reduced to ‘what can be achieved’ as opposed to what might be considered ‘an ideal’ solution. One field researcher talks about how a specifically adapted solutions list was produced as part of the booklet to help people identify potential ideas that might support how they could address their problem(s): *‘No. When, um, when we did the focus groups at one prison site we had a group of men who … some were self-harmers, some were supporters, others were just other prisoners who didn’t self-harm, and they looked at all the materials, and we asked them to generate some solutions. They could identify with all the problems we gave them, they could identify all the emotions and triggers, but they found it difficult to generate solutions’.* It was important to recognise that generating solutions to a particular problem is not easy and nor uncommon. Other studies have shown that individuals who self-harm or who experience severe distress can show elements of attentional fixation (Pratt, 2015). In some cases, they may present with circumstances in which they might be experiencing problems that might not be ‘solvable’ but can be better managed to reduce the level of distress, perhaps similar to people in other situations of crisis (WHO, 2016).

This solution list subsequent formed part of the adaptation process and was used as a prompt to help people think about what options might be available to them when they perceived that ‘nothing could be done’. The process of creating the list of solutions supported the idea that having a ‘positive attitude’ to problem-solving was key to addressing their problems (see Additional file 2):

#### Stage 3: Training staff to deliver the problem-solving skills

The lower level emerging themes around the delivery of the training included: the experiences of receiving training whilst working in an organisation under pressure, the organisation of the training sessions themselves, the format of the training session and how the group sessions worked. This included identifying when was considered a good time to train, and an acknowledgment that problem solving in a prison might not always lead to a problem that could be ‘solved’ but the development of a technique that might help someone to cope better with the circumstances that they are having to deal with. We discuss these in more detail below.

##### Training in an organisation under pressure

Training people to receive new skills in an organisation and working within the constraints of the environment was challenging. During the training period the UK prison service were initiating a series of funding cuts, which resulted in a benchmarking process. In this context, the Government’s intention under the second element of its cost reduction programme was to introduce more efficient ways of working in publicly run prisons, whilst maintaining safety, decency, security and order (see https://publications.parliament.uk/pa/cm201415/cmselect/cmjust/309/30906.htm). This process led to staff redundancies, staff re-grading and staff having to re-apply for their own job. Introducing a new training initiative within this context was challenging and problematic. Many staff felt that staff shortages were prohibitive to training often citing that *‘a lack of time’* and *‘resource’* which forced them into a role which facilitated ongoing ‘crisis management’ on the prison wings: ‘*Again, logistical nightmare. Erm, as it always is in the prison service. Erm, it’s dealing in crisis management*’. This was also reflected in the cancellation of a handful training sessions that meant that training had to be re-arranged often on the day. One staff member refers to the nature of the working in a reactive environment and describes how things change and evolve: *‘I think the training was fine. It was awkward for you because it’s the usual story in here, we’re ever shorter and shorter of staff. You don’t need to tell me anything, I know exactly what it would be like. It would be, you expect such and such, and then such and such happens, and then this evolves and then that changes. It’s not easy.’*

##### Organising the training sessions

The training sessions needed to be flexible and pragmatic to fit into the context of working within the prison environment and as such the research team worked in partnership with each prison site to develop a strategy for how the training could be offered and who could attend the training sessions. Although this was achieved successfully with a greater than expected uptake, *the perception of how staff viewed the training* became an important consideration in how the skills were subsequently utilised. For example, one member of staff talked about how training was offered in a lunchtime: *‘So we don’t … so things can be dropped at the drop of a hat, it was getting people … it were getting bums on seats were the main … were the main problem, then we tried to offer it, err, during a dinner hour, didn’t we, and, err, the enticement of, err, sandwiches … sandwiches and, erm, fizzy drinks.’*

The uptake of training was generated by the use of proactive initiatives in specifically seeking out different staff groups and organisations that worked with the prison to encourage recruitment of staff to the training scheme. This worked well in conjunction with an assigned liaison person within each prison who supported the research team in the practical logistics of organising the training sessions. One field researcher recognises the importance of this contribution and highlights the need for organisational ‘buy in’, collaboration and partnership working to support to enable the facilitation of research: *‘The prison person put a lot of effort into running around for us and helping us with organising people to come to the sessions. That became almost part of that individual’s role. That individual was tasked with helping us to do this particular job. And without that we wouldn’t have managed to get as many individuals trained.’*

##### Format of the training sessions

The format of each training session considering, who, when and where to train in each prison site was negotiated differently at each prison site and was determined by the needs and function of the prison. One prison staff member commented that ‘*…to try and condense that training. I mean, we were lucky that we had fairly small groups. So, we could, we could sort of get that training moved along. If we had bigger groups, then it would have been a lot more difficult.’* The staff member recognised the importance of training in small groups. This was perceived as advantageous because the training could be facilitated in a succinct manner thus supporting the limited availability of staff time. As researchers it was important to recognise that each site was individual and the methodology used to facilitate the process needed to be sufficiently adaptable to deal with these differences whilst maintaining the integrity and fidelity to the training model. For example, one field researcher talks about how the prisons used different approaches to facilitate the delivery of the training: *‘We’ve found huge differences between the prisons. So to all intents and purposes one prison had quite an ad-hoc approach. They were very flexible though. So we trained at one site on a lunchtime. We trained in large ish groups, we trained in small numbers. I even trained individual ACCT assessors. We provided lunch. We trained on induction for staff. So that … they were very helpful in erm, providing us with, with training opportunities that were er, creative in trying to fit around their regime and supporting staff in the training.’* They continue to describe that in other prison sites the approach was different: *‘Erm, in the other prisons, they had a different approach. So they only wanted us to be in the prison and physically around in the prison as well. There was a difference to sort of the sense of us being in the prison, just around in the prison, was that they … we would only train on their lockdown sessions, which was once a month. So the pace was determined by the prison themselves.’*

It became important to fit the training scheme around existing training opportunities (e.g., mandatory planned training session, whereby the prison was on ‘shut down’). It was perceived by staff to be most beneficial when the problem-solving skills training sat alongside other mandatory staff training sessions because staff were more likely to accept that it was part of their role to ‘push this forwards’. One staff member suggests that by incorporating training in this manner it could improve the receptiveness of staff to the new ideas: *‘Perfectly. I think, doing it alongside the case management training is the ideal opportunity. Because they’re the people that you’re expecting to push this forward. And as I say, some of the Senior Officers were very reluctant to sort of take on, on board, new things. Erm, because they get stuck in that routine...’.* We also experienced other competing organisational changes that had perhaps hindered the implementation of the training skills. One staff member talks about how the training coincided with the introduction of the new case manager scheme:*‘...it’s just bad timing. You know, they’ve focused on implementing the new case manager stuff, that’s took precedence over this, you know.’*

Field researchers noted that training was affected also by the function of the prison i.e., whether it housed prisoners awaiting their sentence outcome versus those that offered prisoners a period of resettlement prior to transfer or release into the community. Such factors appeared to reflect in how staff perceived their own roles and staff retention in one prison site a member of healthcare staff reflects on the longevity of staff retention: ‘*But thinking about the nursing staff you have here, I noticed at this prison their turnover of nurses when we were recruiting the nurses to do the training, people would write, I’ve been here two weeks, I’ve been here four weeks, I’ve been here six weeks. We might get eight months. I think I had one person who put, five years, but by and large, at one prison it seemed a very quick turnover. I didn’t get that impression at another prison, so I don’t know … ’.* This finding suggests that training shouldn’t be perceived as a one-off opportunity but as a routine integrated programme of continued booster sessions providing new training sessions for newly employed staff and existing staff to continuously maintain or obtain new skills as employment and loss of staff change overtime.

#### Stage 4: Implementation of the problem-solving skills with prisoners at risk of self-harm

The research team and the staff and the prisoners who received the intervention discussed the feasibility of implementing the intervention. Through the interviews, we primarily wanted to explore why the frontline prison staff had not been able to implement the intervention as had been originally conceived and consider what might need to change so that an implementation mechanism could be used to facilitate the intervention (see Fig. 1). A field researcher recognised that: *we have managed to train a large number of staff, different types of staff. But I think where we’ve hit some barriers is with regards trying to implement their skills actually in practice in some way. So you could say it is feasible to train staff. But then actually getting them to use the skills is a completely different erm area of work really’.*

Delivery of the intervention was primarily promoted using a booklet with the intention of delivering the intervention within a single 30 min session. Whilst this was mainly feasible for the research team (who booked appointments for people to attend in health care) staff (particularly on the wings) suggested that they simply *‘didn’t have the time to sit with someone for this length of time’*. Alternative suggestions for staff to enhance the delivery of the intervention included dividing the booklet into a series of one-page sheets which might only then take a few minutes for each sheet to be described along with some exercises for the prisoner to complete one prison member suggests:*‘Maybe another thing you could have is you could have loose leaf. I’m thinking about your matrix then for something. You could say, okay, maybe this guy’s got excellent skills for … you get the prisoners who can always anticipate the problems. They’ll come up with a million and one problems, but maybe they’re not very good at working out strategies or goals or aims. So, your loose-leaf bit about actually promoting that bit and enhancing that bit. I don’t know, it’s just a thought’.*

Staff found it difficult to implement the intervention particularly where the turnover of prisoners was great and previously tested and tried methods used by staff took precedence over using the new skills. One member of staff speaks of the operational running of the prison referring to the function of a local prison which had a high turn-over of prisoners providing little continuity and opportunity for them to support prisoners: *Erm, I used different … well, I used my own. I’ve got my own methods and things, you know. ‘You know, err, we are a local jail, we serve the courts, we’ve got to … we’ve got to ship them out, that’s my role at the minute. Yeah, that’s the problem, yeah, that’s the only problem, we can’t … we can’t really keep hold of them or … or trap them as such*.’

##### Engagement with the intervention and impact of the prison environment

Engagement with the intervention by prisoners was affected by different factors. One prisoner explained that he did not engage with the intervention at all, dismissing it as *‘a load of rubbish’*. He explained that he only agreed to be involved in the study because he was ‘*on basics*’ at the time and so was confined to his cell much of the time and had many of his privileges removed. Taking part in the study was an opportunity to leave his cell. When probed, he offered reasons for his lack of engagement, including the very fact of being in prison is depressing and then being asked to look at their own depressive feelings can result in feeling more depressed, rather than helping, as he explained: *When you’re in here you’re already on a downer, aren’t you? Looking at something about depression, you’re even more depressed, to be honest’.*

The perception of what the intervention is about appears to play a key role in whether someone will want to engage. The personal circumstances of each individual prisoners impacted on whether they felt they had the capacity to engage with the intervention. One prisoner said:*‘I’ve got a lot in my head, yeah. I’m on trial next Monday. Yeah, I’ve got a lot on, yeah. My nana’s not very well and I’m stuck in here.’* Engagement needed to be carefully timed to ensure an individual’s readiness and ability to take part in the intervention. One field researcher recognised this: *Yeah, so there’s a sense that after you’ve got an opportunity, a window opportunity whether someone is going to be keen and want to engage with you and then after that, for whatever reason, they’re not prepared to come back or they’ve had enough of it or they haven’t gone any further with the booklet perhaps and we don’t see them again. I think it’s interesting for the model for future to think about the … how many sessions might be a good amount to, engage with people and what that might look like.*

Some participants described the challenges associated with being in the prison environment as having *‘a central lack of control’* over the means through which they might perceive that they could resolve their problems: *Yeah, you’ve got no control over them, the problems don’t go away, they just get worse and eat away at you. Until you can deal with the problem it’s still going to eat away at you, no matter if you go and look at a magazine, the problem’s still there and as soon as you’ve read the magazine that problem’s back in your head because there’s nothing to do in here. You don’t get out much so your problems are always there.’* Thus, for some participants, ‘problem solving’ implied fixing them and sorting them out but this was not possible in a prison context because prisoners have restricted freedoms which limit their ability to actively resolve their problems.

##### Engagement helped by prior exposure to other courses

Engagement with the skills seemed to be enhanced by prior experience of self-help courses and the prisoner’s level of self-awareness. For example, although one prisoner had recently split up with his girlfriend, he still engaged with the intervention. What appeared to enable him to engage was his capacity for self-reflection. He explained that ‘*it weren’t too bad*’ filling the booklet and that completing the booklet came ‘*pretty easy to be honest’. ‘I think I know what my problems are kind of thing’*. This response suggests that the prisoner already had a certain degree of insight into what his problems were, which made completing the booklet easier. Later in the interview, he also explained that he had ‘*done Thinking Skills Programme before and some of it is similar, so it is just taking easy little steps and then trying to progress and using it to your advantage, that’s going to be a major one*’.

Thus, it may be that previous exposure to similar interventions made engagement with the intervention easier as it improved this participant’s ability to self-reflect, or that participants who have higher levels of self-awareness are more likely to engage with these sorts of interventions in the first place (or both). Similarly, another participant was going through a divorce but engaged with the intervention ‘*because I’ve got problems and I needed help*’.

##### Intervention mechanisms how did it work?

The process of self-reflection changed participant’s thoughts and behaviour in a range of different ways. Overall, self-reflection and gaining insight into their problems enabled participants to manage their behaviour and cope more effectively. However, participants recounted differing degrees of success with enacting the skills that completing the booklet sought to equip them with and the intervention appeared to work in subtly different ways for each participant. Some participants seemed to gain benefits in dealing with a specific problem or issue – their narratives focused largely on explaining how a technique had helped them. One participant appeared to gain a wide range of skills and techniques from the intervention and was able to teach and support others with these skills. Finally, some participants were less secure and certain about their abilities to utilise the skills to cope with or manage problems this prisoner pinpointed that it had been the process of “*working the problems out one by one*” that was helpful “*instead of having all the problems at once*”. He used the analogy of a book to explain how working on one problem at a time had prevented him from feeling overwhelmed by his problems: *‘The best way I can describe it at the moment is, that’s a book. Each one of them chapters in the book. You’ve got to get through one problem before you can start on another one. If you try and work them all out in bits at the same time, it doesn’t work. You lose where you are. And then you end up going back to step one, which means you get emotional, you get your behavioural problems come back again. So to break it down and then go down each one*.’

He explained that using this technique had enabled him to effectively prepare and deliver a presentation to a group of nine people, something he had never managed before: *‘Erm I think for most of the people I’ve seen, um, there’s been an element of introducing some sort of coping strategies in there, um, so they acknowledge that yes one, this is a problem I can sort out and I also have these other problems I can’t sort out, so I’m going to apply the coping strategies to those and just do the ones I can’.* Other prisoners described using the visual imagery of putting his problems in a box and reading and watching television to enable him to relax: ‘*Like I say, put the problem in the box outside your door. A visible box outside your door, put all your problems in there because you can’t get to them because the door’s locked*.’

##### Perceived impact of the intervention on self-harm

Overall, self-harm appeared to decrease over time, but our conclusions are limited due to the lack of a comparable control group. Individual reports from those participants taking part showed 32/48 people self-harming in the 3 months prior to baseline, with only nine people reporting self-harm immediately following the intervention. One prisoner talked about how this felt:*‘Since I’ve started this … this booklet and doing the bit of education, I’ve only self-harmed once*: the interviewers asks: *‘Mmhmm, okay. And how much would you say you were doing it before that?, the prisoner responds: ‘About two maybe three times a week*.’ *I feel a lot better, because I know that if I’ve got a problem I can learn how to work through it, where before I just used to cut myself just to get rid of the pain*.’

##### Sustainability of the intervention delivery

It was clear that using staff to implement the intervention in a highly pressured environment was not feasible. Alternative ideas about how the scheme could be implemented were discussed by prisoners one commented: *‘So perhaps that’s also an argument for extending the problem-solving training, to offer it as a class, because … you say there seems to be quite a few prisoners who are keen to use it in conjunction with their classes, in conjunction with the information desk work. But because they’ve not self-harmed they don’t have access to it. At one prison we offered the training but I think it’s a lot to expect the prisoners to come up, attend for one hour and be comfortable in using it’.* Another suggested the benefit of peer support: *The problem orientation worksheet, if you’ve got a mentor available to erm go through that and explain what everything means, and discuss it a little bit, then that’s...that’s great. Erm as I alluded to before, if you get someone like myself, I’d know what those meant, and I’d just tick yeah, yeah, agree or disagree.’* And also having the availability of someone (other than staff) to support on the wings *‘The booklet has been quite helpful, it’d be better if there was someone, like, to help us go through the booklet with me on the wing, when I’ve got time … .but the workers don’t seem interested in it and the staff can’t be … they haven’t got time to, but the things I’ve been doing is writing down my agreements and disagrees on that one that I’ve put down*.’

Later in the interview he expanded on these comments to explain that it was not just helpful to complete the booklet he would also have liked advice and support on dealing with the problems identified through completing the booklet:*‘As I say, it just needs somebody to be there if on an evening, or something, you’ve got a problem, you know someone who can go to and say, look, I’m having this problem with this, any advice on it? ‘Erm, supported by a peer mentor, which … which is fine, maybe that’s … that’s the way forward, I don’t know, but it … it did seem that like we’re being … that was just recovering stuff that we … we have already covered in the past’.*

## Discussion

The aim of this study was to: (i) assess the feasibility and acceptability of implementing a problem-solving training package for frontline prison staff, and (ii) cascade the skills to prisoners at risk of self-harm. Adaptation of the materials was imperative to developing an intervention that was perceived by the participants as something that they could relate to. Examples of other co-production activities in the promotion of healthcare have also found that this engagement is paramount to its success (Lorig et al., 1999). These findings concurred with this current study; whereby involving prisoners in the process provided a catalyst generating a ‘bottom up’ approach to enhance and support the engagement with frontline prison staff.

Training was organised with staff who were working under pressure with limited resources. Staff only received a one-hour training session which limited the implementation of the skills and compounded the other organisational constraints of working within the prison. Research on prison environments and the culture of the organisation support that when you have an inexperienced workforce with staffing shortfalls and low retention that any training opportunities can be under mind (Liebling & Arnold, 2004; Taylor & Cooper, 2008).

In an ideal scenario, one would want to wait until a ‘steady state’ was achieved within an organisation before trying to implement change. The timing of the project was however pre-determined by the research funding (as opposed to the other way around). The training was delivered in partnership and collaboration with the prisons, using a pragmatic, proactive and flexible approach we managed to train numbers of staff well exceeding our original target of 125 staff. Within the four prison sites we trained staff using different strategies, most well received was when training was embedded within other organisational training initiatives (e.g., within safer custody) because it was more likely to be *perceived as training that was mandated* to complete and with that was an expectation that staff had some responsibility to take the role forwards.

Differences in staff turnover across our four prison sites supported the suggestion that staff training needed to be a continuous process that would seek to provide skills for new staff joining the prison service but also provide an opportunity for ‘booster sessions’. Turnover of prisoners and staff at our local prison sites (A & B) were considerably greater than our resettlement site (D). Such findings may provide insight into the design of future research studies that might seek to measure the impact on outcomes of effectiveness.

Prisoner turnover, staff resources and the changing dynamics of the prisoner population hindered the intervention delivery by staff. Engaging professionals as co-productive partners was difficult and time consuming in this context. Examples of staff doing ‘what they have always done’ – or inconsistently applying new found skills has also been reported elsewhere (Epstein, Alper, & Quill, 2004). Delivery of the intervention with prisoners at risk of self-harm were conducted, in the main, by the research team. The natural diversity amongst prisoners meant that not all elected to engage with the intervention for a variety of reasons. There was a clear interplay between the prison environment and the level of engagement with the intervention. This finding reflects the complexity of delivering interventions in criminal justice settings. This further supports the need for adaptation of future co-produced training initiatives (see http://personcentredcare.health.org.uk/resources/development-of-e-learning-module-clinicians/).

Prisoners struggled to engage with the intervention if they were experiencing depression, significant family life events or were at decision and/or crisis point in their prison journey. Not everyone we saw was ready to engage with the intervention. Future evaluations may need to consider the inclusion criteria to include a measure of ‘readiness to change’ (Rollnick, Heather, Gold, & Hall, 1992) and personal circumstances which might impact on problem-solving processes.

Some prisoners interpreted the intervention as seeking to help them ‘solve’ their problems. In a practical sense, some felt that their problems were ‘too big’ to be amenable to change in this way reflecting this idea promoted by the World Health Organisation as ‘problem management’ might be a more adept phrase. The culture of the prison environment and inter-play between the prisoners and staff relationships are also crucial in how any such skills are delivered by staff and received by the prisoners Research by Crewe refers to the idea of ‘soft power’ which presents a complex relationship between staff who are required to support prisoners to act in resolving their own problems as part of the rehabilitative process and policy guidance whilst maintaining obtaining security information on prisoners which might hinder and facilitate their progression through the prison system. Officers provide the first point of call for links to offender managers and outside agencies and for information about offending behaviour courses and increasingly complex sentence conditions. For prisoners on long and indeterminate sentences, ‘progression’ through the system is as vital a part of the prison experience as food, visits and mail (Crewe, Liebling, & Hulley, 2011) and arguably they themselves need to play a role in supporting the prisoner in the process of problem-solving.

Most prisoners who engaged with the intervention felt that it had enabled them to become aware of, and better identify and name their emotions, and some felt that the intervention has enabled them to manage their emotions and behaviour more effectively. One prisoner utilised a wide range of techniques taught by the intervention and had supported other prisoners to use these techniques. Familiarity with the problem-solving skills was advantageous to those who engaged with the process. Prisoners showed clear mechanisms of self-reflection and visualisation techniques, leading in some cases to anecdotal evidence to reduce self-harm. One prisoner reported that because the intervention had enabled him to break his problems down into ‘smaller chunks’ it had reduced his tendency to self-harm as he could know deal with his emotional difficulties in a different way. Our wider evaluation of this data showed overall that incidence of self-harm reduced. Whilst it is inappropriate to attribute any statistical significance to these findings further, exploration is required (Perry et al. 2019 in press).

Given that, the study failed to provide an implementation mechanism for the intervention feedback from staff and prisoners about how the intervention could be implemented were crucial to consider in how to develop the study findings. The first, suggested that prisoners could be educated in groups through the commission of education providers. Current educational provision in UK prisons are contracted through a tendering service within prison regions. Within this remit, this would mean that a problem-solving intervention would be provided at least until the end of a contracting period thus guaranteeing the sustainability of the scheme. The second, proposed the development of a prisoner peer-led scheme whereby prisoners would be trained to pass the skills onto their peers. Both suggestions have merit and require further exploration in the delivery of the intervention.

## Implications for practice

A number of lessons can be learnt and/or implied about how to deliver and implement training skills for prison staff the findings support that: (i) training needs to be an ongoing sustainable process that becomes part of what the prison does as opposed to a one off session, (ii) training should be incorporated into existing mandated training for staff, (iii) training should be available on induction courses for new staff joining the prison as well as part of an ongoing strategy to maintain the skills of staff who have been within the services for some time, (iv) the timing and implementation of any new initiative within the prison site should be carefully timed to ensure where possible that it doesn’t coincide with any other changes that staff are meant to deal with, and (v) intervention delivery needs to suit the needs of staff in a brief format that can be delivered in a few minutes of repeated support throughout the working week.

## Conclusions

The study was established first to assess the adaptation, feasibility and implementation of a problem-solving community-based intervention for staff who were trained to deliver the skills with prisoners at risk of self-harm. Adaptation of materials was well received, despite large numbers of staff being trained, it was deemed unfeasible for them to deliver the skills to those prisoners at risk of self-harm. Some prisoners demonstrated clear benefits from taking part in the intervention whilst others found it difficult to engage due to a variety of contextual issues. Alterative implementation mechanisms are important to consider in the future development of the scheme. These could include implementation via educational providers and or the development of a peer-led scheme.

## Additional files

Additional file 1: Examples of the problem-solving booklet. (DOCX 69 kb)

Additional file 2: Example Solution List. (DOCX 14 kb)

## Data Availability

Participant level data, the full data set and statistical codes are available from the corresponding author.

## Abbreviations

ACCT: Assessment Care in Custody and Teamwork
CBT: Cognitive Behavioural Therapy
HMP: Her Majesty’s Prison
HMPPS: Her Majesty’s Prison and Probation Service
PST: Problem Solving Training
WHO: World Health Organisation

## References

[CR1] Alford John, Yates Sophie (2015). Co-Production of Public Services in Australia: The Roles of Government Organisations and Co-Producers. Australian Journal of Public Administration.

[CR2] Batalden Maren, Batalden Paul, Margolis Peter, Seid Michael, Armstrong Gail, Opipari-Arrigan Lisa, Hartung Hans (2015). Coproduction of healthcare service. BMJ Quality & Safety.

[CR3] Collinson M, Owens D, Blenkiron P, Burton K, Graham L, Hatcher S, Farrin A (2014). MIDSHIPS: Multicentre intervention designed for self-harm using interpersonal problem-solving: Protocol for a randomised controlled feasibility study. Trials.

[CR4] Crewe B, Liebling A, Hulley S (2011). Staff culture, use of authority and prisoner quality of life in public and private sector prisons. Australian & New Zealand Journal of Criminology.

[CR5] D’Zurilla TJ, Goldfried MR (1971). Problem solving and behavior modification. Journal of Abnormal Psychology.

[CR6] Daunic AP, Smith SW, Garvan CW, Barber BR, Becker MK, Peters CD, Naranjo AH (2012). Reducing developmental risk for emotional/behavioral problems: A randomized controlled trial examining the Tools for Getting Along curriculum. Journal of School Psychology.

[CR7] D'Zurilla TJ, Maydeu-Olivares A, Kant GL (1998). Age and gender differences in social problem-solving ability. Personality and Individual Differences.

[CR8] Epstein R, Alper B, Quill T (2004). Communicating evidence for participatory decision making. JAMA.

[CR9] Evans K, Tyrer P, Catalan J, Schmidt U, Davidson K, Dent J, Thompson S (1999). Manual assisted cognitive behavioural therapy in the treatment of recurrent deliberate self harm: A randomised controlled trial. Psychological Medicine.

[CR10] Forrester A, Slade K (2014). Preventing self-harm and suicide in prisoners: job half done. The Lancet.

[CR11] Hawton K, Linsell L, Adeniji T, Sariaslan A, Fazel S (2014). Self-harm in prisons in England and Wales: an epidemiological study of prevalence, risk factors, clustering, and subsequent suicide. Lancet.

[CR12] Hawton K, Witt KG, Taylor Salisbury TL, Arensman E, Gunnell D, Hazell P, ... van Heeringen, K. (2016). Psychosocial interventions for self-harm in adults. Cochrane Database of Systematic Reviews, Issue 5. Art. No.: CD012189. DOI: 10.1002/14651858.CD012189. 10.1002/14651858.CD012189 PMC8786273 27168519

[CR13] HMIP (1999). Suicide is everyone’s concern: A thematic review by HM chief inspector of prisons in England and Wales.

[CR14] Liebling, A., & Arnold, H. (2004). *Prisons and their moral performance: A study of values, quality, and prison life*. Oxford: Clarendon Press.

[CR15] Linehan MM, Camper P, Chiles JA, Strosahl K, Shearin E (1987). Interpersonal Problem-Solving and Parasuicide. Cognitive Therapy and Research.

[CR16] Lorig KR, Sobel DS, Stewart AL, Brown BW, Bandura A, Ritter P, Gonzalez VM, Laurent DD, Holman HR (1999). Evidence suggesting that a chronic disease self-management program can improve health status while reducing hospitalization: a randomized trial. Medical Care.

[CR17] McLeavey BC, Daly RJ, Ludgate JW, Murray CM (1994). Interpersonal problem-solving skills training in the treatment of self-poisoning patients. Suicide Life Threat Behav.

[CR18] Ministry of Justice (2016). Safety in custody bulletin in England and Wales: Deaths in prison custody to June 2016.

[CR19] Pawson R, Tilley N (1997). Realistic evaluation.

[CR20] Perry A, Waterman M, House A, P D (2015). Problem-Solving Training for Suicidal Prisoners. The Prevention of Suicide in Prison: Cognitive Behavioural Approaches.

[CR21] Perry A. E., Waterman, M. G., House, A. O., Wright-Hughes, A., Greenhalgh, J., Farrin, A., Wright, N. (2019). Problem-solving training: assessing the feasibility and acceptability of delivering and evaluating a problem-solving training for frontline prison staff and prisoners who self-harm. British Medical Journal Online, in press. 10.1136/bmjopen-2018-026095 PMC6797432 31585968

[CR22] Pestoff V (2009). Towards a paradigm of democratic participation: citizen participation and co-production of personal social services in Sweden. Annals of Public and Cooperative Economics.

[CR23] Pollock L, Williams J (2011). Effective problem solving in suicide attempters depends on specific autobiographical recall. Suicide and Life Threatening Behaviour.

[CR24] Pratt D, Tarrier N, Dunn G, Awenat Y, Shaw J, Ulph F, Gooding P (2015). Cognitive Behavioural suicide prevention for male prisoners: A pilot randomised controlled trial. Psychological Medicine.

[CR25] Radnor Z, Osborne S, Kinder T, Mutton J (2014). Operationalizing Co-Production in Public Services Delivery: The contribution of service blueprinting. Public Management Review.

[CR26] Rollnick S, Heather N, Gold R, Hall W (1992). Development of a short 'readiness to change' questionnaire for use in brief, opportunistic interventions among excessive drinkers. British Journal of Addictions.

[CR27] Sijbrandij M, Farooq S, Bryant RA, Dawson K, Hamdani SU, Chiumento A, van Ommeren M (2015). Problem Management Plus (PM+) for common mental disorders in a humanitarian setting in Pakistan; study protocol for a randomised controlled trial (RCT). BMC Psychiatry.

[CR28] Taylor P, Cooper C (2008). ‘It was absolute hell’: Inside the private prison. Capital and Class.

[CR29] Walker Tammi, Shaw Jenny, Turpin Clive, Reid Catherine, Abel Kathryn (2017). The WORSHIP II study: a pilot of psychodynamic interpersonal therapy with women offenders who self-harm. The Journal of Forensic Psychiatry & Psychology.

[CR30] WHO (2014). Preventing suicide: a global imperative.

[CR31] World Health Organization (2016). Problem Management Plus (PM+): Individual psychological help for adults impaired by distress in communities exposed to adversity. (Generic field-trial version 1.1).

