# Supplementary material for: Implementation of a problem-solving training initiative to reduce self-harm in prisons: a qualitative perspective of prison staff, field researchers and prisoners at risk of self-harm
Source: Health Justice. 2019 Jul 31;7:14. doi: 10.1186/s40352-019-0094-9 (PMC6717963; doi:10.1186/s40352-019-0094-9)
Supplement: Supplementary file 1 — Examples of the problem-solving booklet. (DOCX 69 kb) [file 40352_2019_94_MOESM1_ESM.docx]

Additional file 1: Examples of the problem-solving booklet

**RECOGNISING HOW YOU FEEL – CAN YOU CHANGE HOW YOU BEHAVE WHEN A PROBLEM OCCURS?**


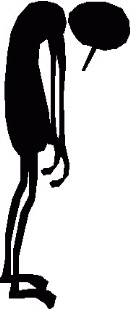


*NEGATIVE THOUGHTS:*

*I can’t be bothered.*

*What’s the point?*

*Nothing can be done.*

*
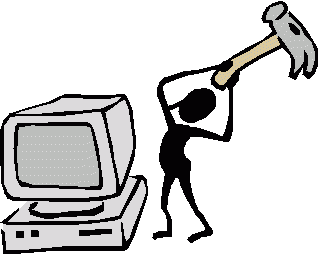
*

*BEHAVIOUR*

*Kicking off, breaking things,*

*withdrawing, laying low.*

*RECOGNISE YOUR FEELINGS*

*Angry – frustrated, anxious*

*Panicky- hopeless - helpless*

*
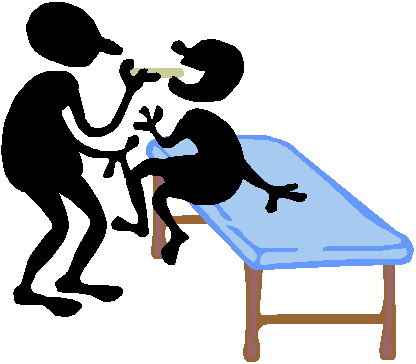
*

*PHYSICAL SYMPTOMS*

*Feeling ill, tense muscles,*

*stomach aches, can’t sleep,*

*heart pounding, sweaty palms*

**Learn to listen to yourself- Recognise the sign and triggers**
