# Supplementary material for: Implementation of a problem-solving training initiative to reduce self-harm in prisons: a qualitative perspective of prison staff, field researchers and prisoners at risk of self-harm
Source: Health Justice. 2019 Jul 31;7:14. doi: 10.1186/s40352-019-0094-9 (PMC6717963; doi:10.1186/s40352-019-0094-9)
Supplement: Supplementary file 2 — Example Solution List. (DOCX 14 kb) [file 40352_2019_94_MOESM2_ESM.docx]

Additional file 2: Example Solution List

| Try out strategies to stop me from getting me wound up, relaxation or deep breathing |
| --- |
| Don’t brood about the past or future, focus on the here and now. |
| When I get wound up walk away from the situation |
| Talk to someone on the wing before I lash out, count to ten |
| Keep myself away from people who bother me |
| Ask prison staff whether I can be moved onto another wing away from a bully |
| Speak to the a prison listener or the Samaritans to talk about my problems |
| Speak to prison Chaplaincy to discuss your problems or find your faith |
| Keep busy and try to fit in to prison life |
| Learn to distract yourself – read, make something, art work, go to the gym, do exercises in your cell. |
| Write about your experiences |
| Contact the prisoner information desk on your wing |
| Find out what education is available in the prison |
| Find out what work opportunites are available in prison |
| Book a phone call home |
| Arrange a visit for family or a friend |
| Contact DARS - Drug and Alcohol Services for prison and probation services |
| Contact health care |
| Find out about the Inreach team for Mental Health – discuss the possibility of counselling |
